# Septic Arthritis of the Pubic Symphysis in an Adolescent Athlete: A Rare and Atypical Presentation

**DOI:** 10.7759/cureus.105787

**Published:** 2026-03-24

**Authors:** Ali Tariq, Muhammad Mubeen Bashir, Mahrukh Azhar, Eswara Murthy, William Gaine

**Affiliations:** 1 Orthopaedic Surgery, Sligo University Hospital, Sligo, IRL; 2 Palliative Care, Mayo Hospice, Castlebar, IRL

**Keywords:** dalvabancin, osteo-myelitis, pubic symphysis, septic arthritis, staph aures

## Abstract

Septic arthritis of the pubic symphysis is an uncommon and often overlooked diagnosis, particularly in adolescents without clear risk factors. We report the case of a 20-year-old male who presented with atypical groin and hip pain following recent participation in a hurling match, and was ultimately diagnosed with septic arthritis of the pubic symphysis. The clinical presentation was subtle and non-specific, lacking typical signs of hip joint involvement. MRI imaging was crucial in establishing the diagnosis, and the patient responded well to targeted intravenous antibiotics. The patient received 14 days of intravenous antibiotics followed by dalbavancin and achieved full recovery by six weeks. This case highlights the importance of considering pubic symphysitis in the differential diagnosis of groin and thigh pain in athletic adolescents.

## Introduction

Septic arthritis of the pubic symphysis is a rare entity, accounting for less than 1% of all cases of osteomyelitis [1]. It has been well-documented in children, individuals with a history of intravenous drug use, and elderly patients following genitourinary procedures. However, it may also occur in otherwise healthy individuals, with strenuous athletic activity recognized as the sole predisposing factor [2]. The condition poses a diagnostic challenge due to its nonspecific presentation, often mimicking more common musculoskeletal or abdominal disorders [1]. This uncommon cause of febrile illness in athletes should therefore be considered, particularly as localizing clinical signs may be subtle or absent at initial presentation [3].

Typical clinical features include fever and abdominal, pelvic, or groin pain that worsens with standing, walking, or hip movement, frequently accompanied by painful claudication [4]. While both septic and inflammatory arthritis of the pubic symphysis may present with similar symptoms, septic arthritis usually manifests more acutely and is characterized by pronounced pain and systemic fever [1,5,6]. Prompt recognition and treatment are crucial to prevent complications, as diagnostic delays of several days to weeks have been reported due to overlapping musculoskeletal symptoms [1].

We present a rare case of pubic symphysis septic arthritis in an otherwise healthy young athlete with an atypical clinical presentation. This case highlights the diagnostic value of MRI and microbiological testing in confirming the condition, and demonstrates that timely, targeted antibiotic therapy can result in full recovery without the need for invasive procedures.

## Case presentation

A 20-year-old previously healthy male presented to the emergency department with a 3-day history of worsening right hip pain and limp following a hurling match. There was no history of trauma, recent viral illness, or sexual activity. Five days before presentation, he experienced mild groin discomfort that progressed to severe pain and restricted mobility. He appeared flushed and was febrile with spiking temperatures, requiring crutches for ambulation due to pain.

On examination, there was no clinical evidence of right hip irritability. However, straight leg raise on the left side and left hip flexion reproduced pain in the right thigh, particularly along the adductor region. There was no tenderness in the abdomen or groin on palpation.

**Figure 1:**
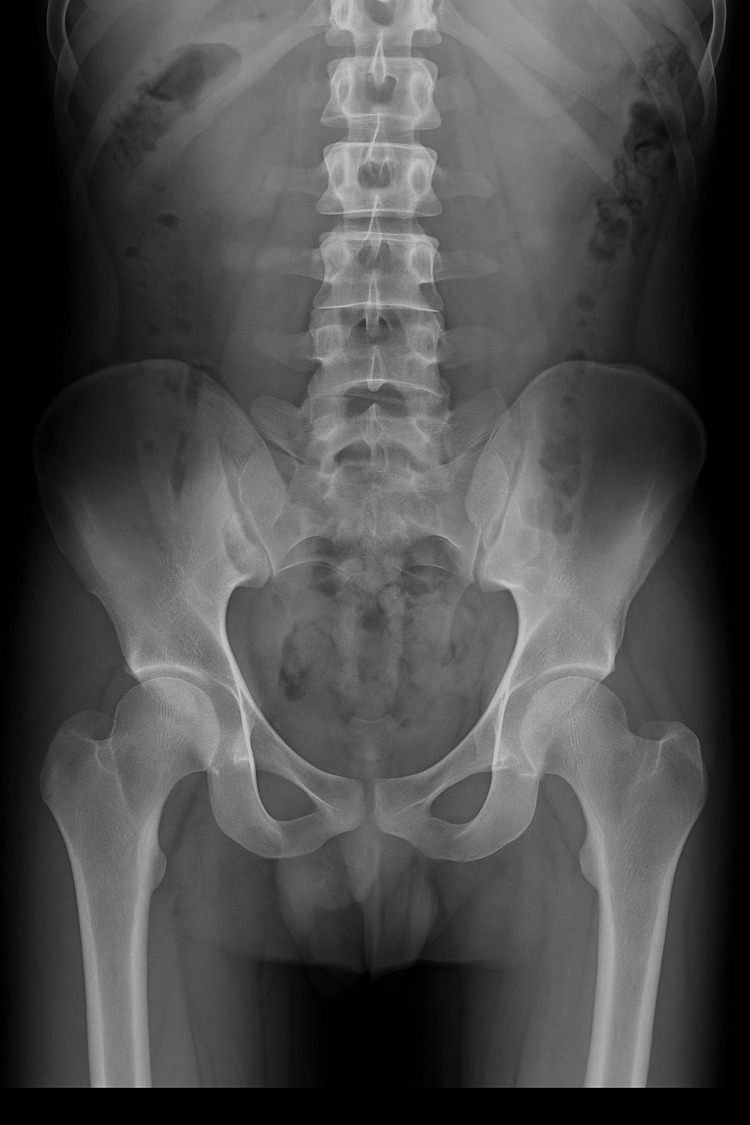
X-ray on the day of presentation No obvious bone pathology is noted.

Laboratory investigations demonstrated elevated inflammatory markers and leukocytosis (Table 1). CRP was 332, WCC 14.5, and Hb was 11.9 on the day of admission. Blood cultures were obtained, and the patient was started on intravenous flucloxacillin for a presumed musculoskeletal infection. Initial radiographs of the hips, pelvis, and femurs were unremarkable (Figure 1).

**Table 1:** Laboratory investigations

Parameter	On Admission	Day 3	Day 5	Day 21	Reference Range	Interpretation
White blood cell count (×10⁹/L)	14.5	10.7	8.6	7.6	4.0–11.0	Elevated on admission, normalized during recovery
Hemoglobin (g/dL)	11.9	11.6	12.7	13.9	13.0–17.0	Mildly reduced initially, improved over time
Platelet count (×10⁹/L)	170	195	386	245	150–400	Normal to mildly elevated during treatment
C-reactive protein (mg/L)	332	365	134	3	<10	Markedly elevated initially, declined progressively with treatment
Creatinine (µmol/L)	95	85	86	71	60–110	Within normal limits throughout admission
Urea (mmol/L)	5.4	5.9	5.2	4.7	2.5–7.0	Within normal range
eGFR (mL/min/1.73 m²)	>90	>90	>90	>90	>90	Stable renal function
Blood cultures	Staphylococcus aureus	—	—	—	—	Positive on admission, cleared after treatment; sensitive to vancomycin and dalbavancin

**Figure 2:**
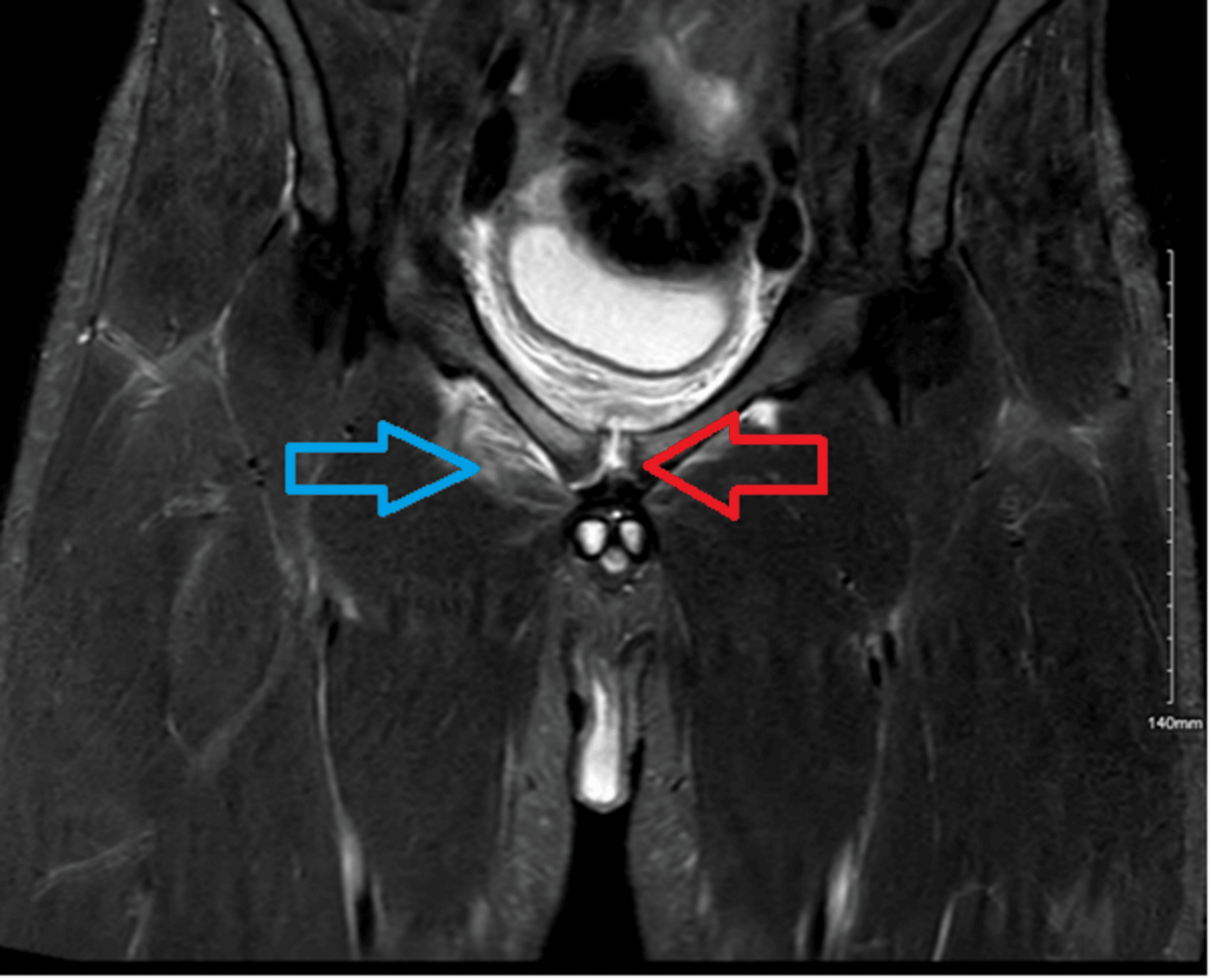
MRI scan Small foci of septic fluid are visible in the vicinity of the pubic symphysis. The blue arrow denotes edema and collection in the right obturator internus, whereas the red arrow shows foci in the pubic symphysis.

**Figure 3:**
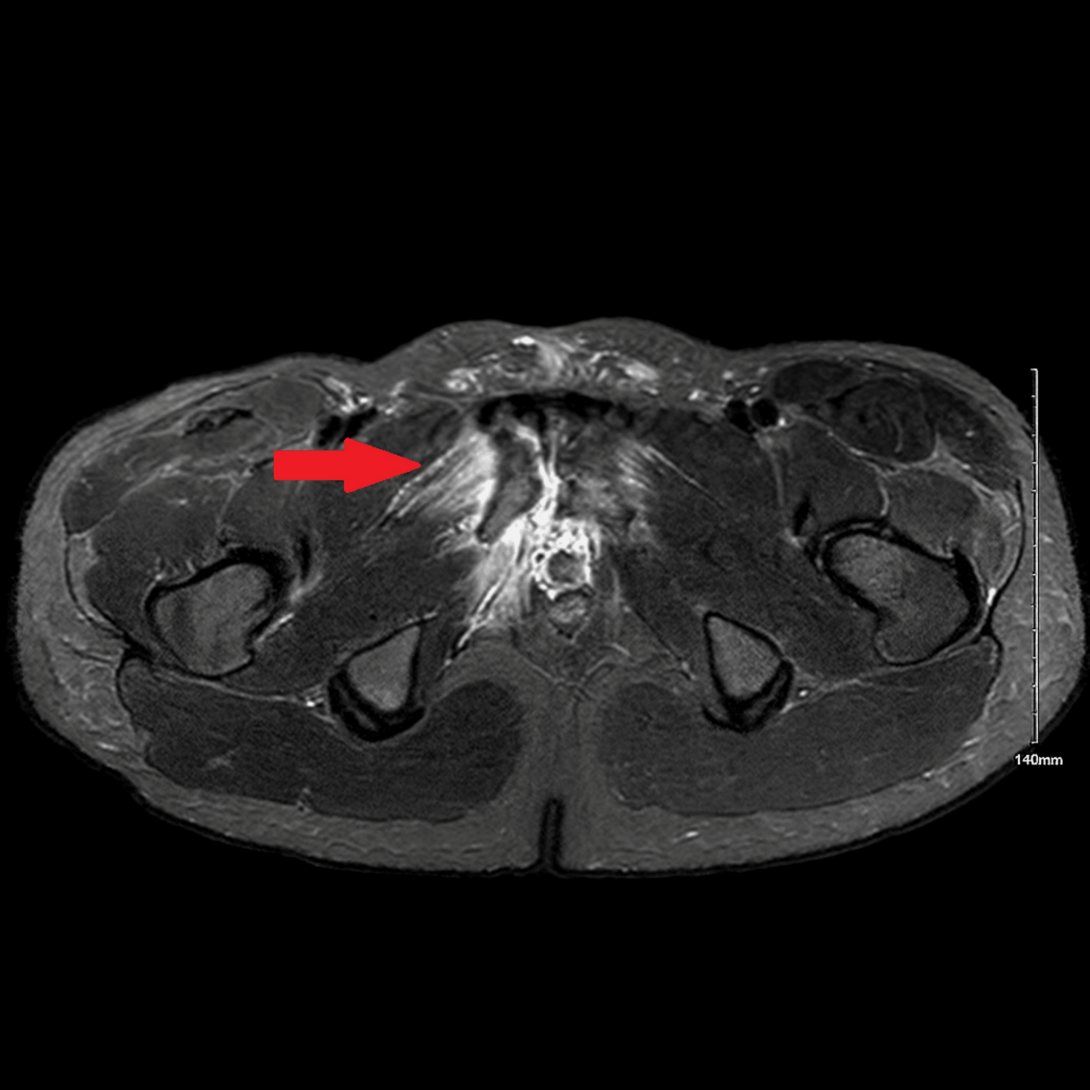
MRI axial view The arrow denotes edema and foci of fluid in the vicinity of the pubic symphysis and the right obturator internus muscle.

**Figure 4:**
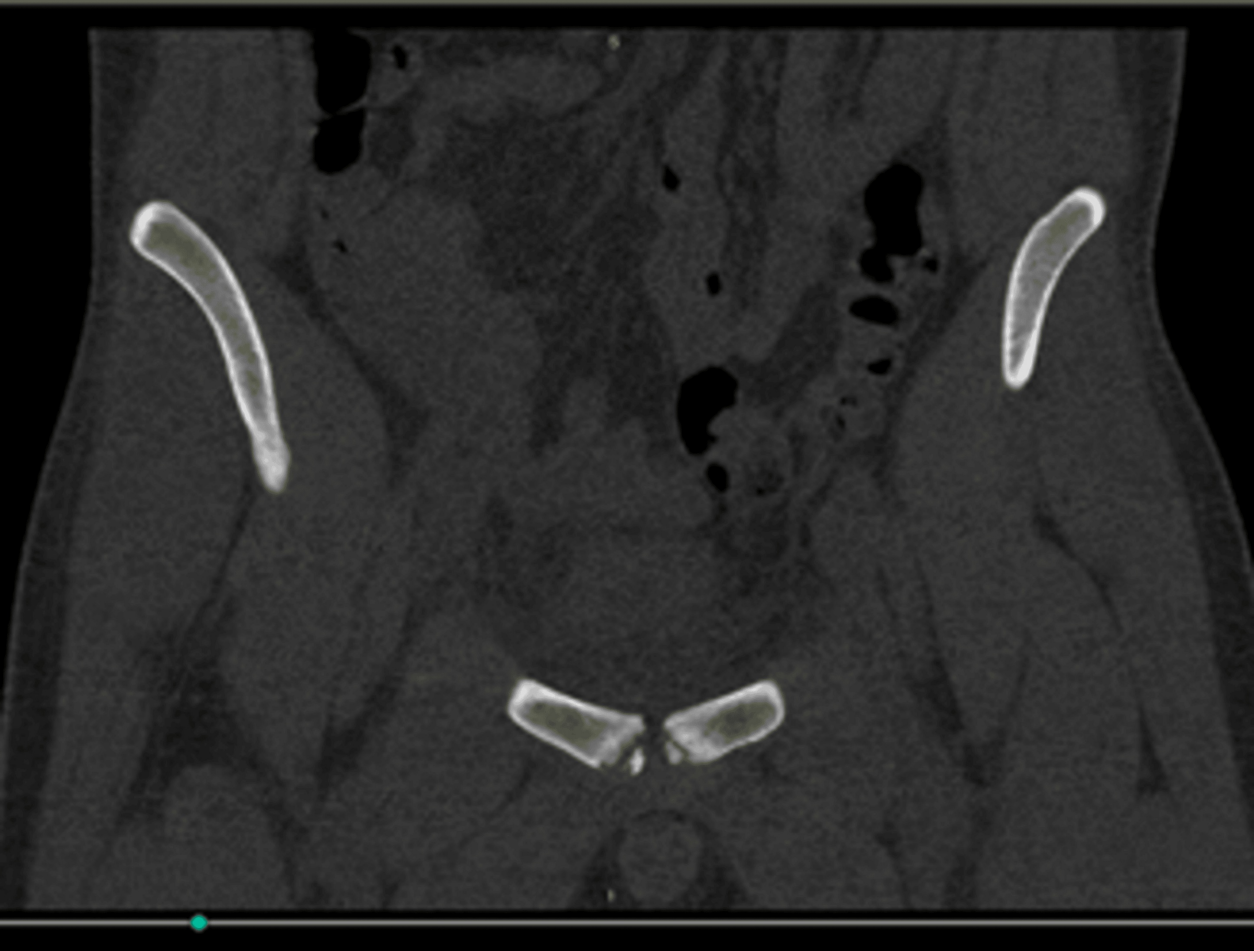
CT scan showing evidence of osteitits pubis Foci were insufficient in size to allow image-guided drainage.

Due to a lack of clinical improvement, an urgent MRI scan of the pelvis was performed on the same day, revealing small fluid collections consistent with pus formation around the pubic symphysis (Figures 2, 3). The case was reviewed with the pelvic orthopedic, microbiology, and interventional radiology teams. A CT scan confirmed small pockets of fluid; however, due to their limited size and location, image-guided aspiration was deferred (Figure 4).

Subsequent blood cultures grew *Staphylococcus aureus* sensitive to vancomycin and dalbavancin. The patient was transitioned to intravenous vancomycin, resulting in gradual clinical improvement. Inflammatory markers declined, and pain symptoms improved. An echocardiogram was obtained, which did not reveal any concerning evidence for infective endocarditis. During his two-week hospital stay, mobility steadily improved. He was discharged on dalbavancin and followed up in the outpatient clinic.

**Figure 5:**
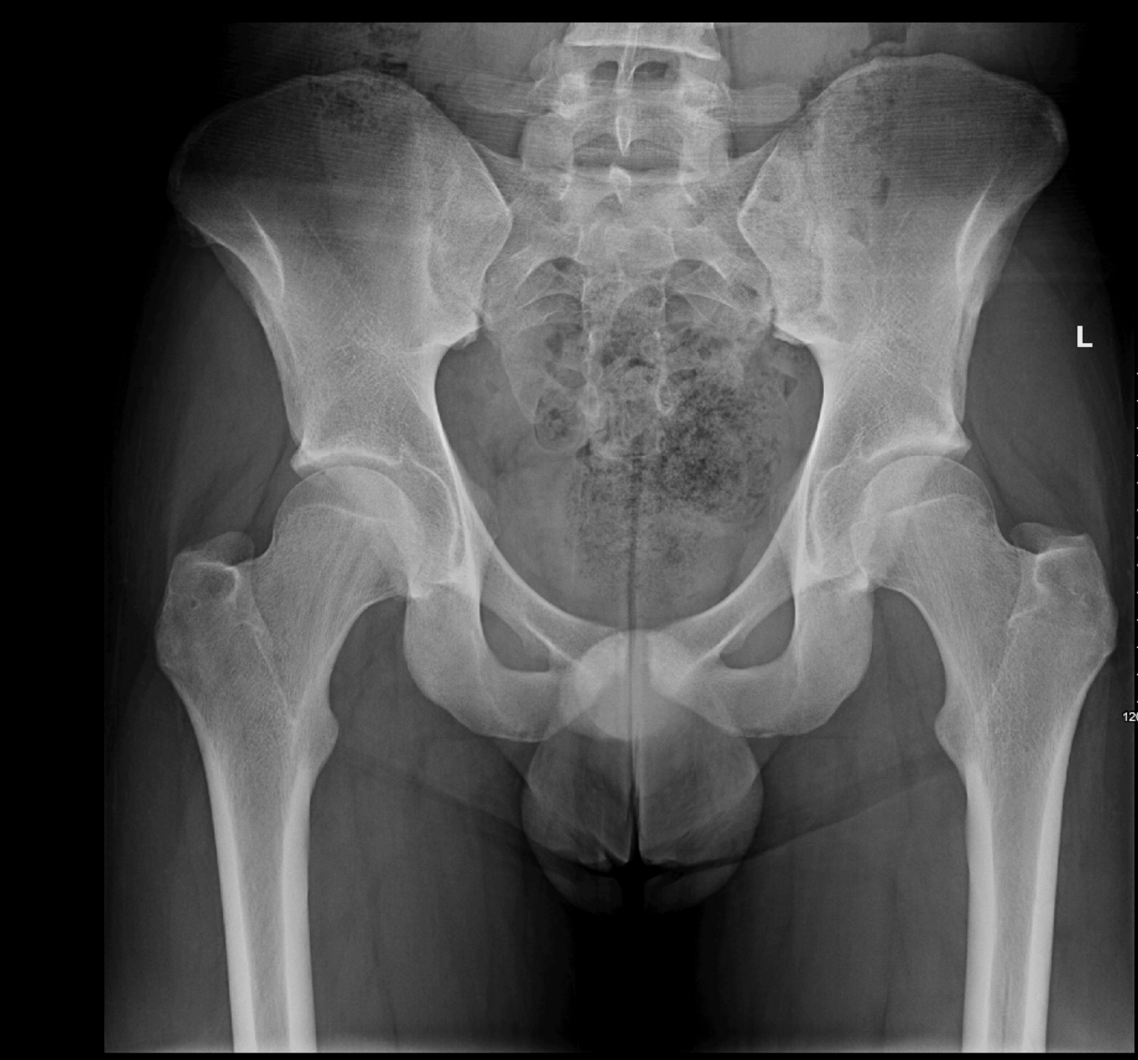
X-ray at the one-year follow-up Stable appearance of the osteitis pubis

At the 2-week and 6-week follow-up visits, he reported marked clinical improvement. By six weeks post-infection, he had returned to full mobility with minimal pain and no limp or significant functional limitations. At the one-year follow-up, the patient had achieved complete functional recovery with no residual symptoms or evidence of recurrent infection (Figure 5).

## Discussion

Septic arthritis of the pubic symphysis is a rare diagnosis, particularly in adolescents without identifiable risk factors, and remains a challenging entity to recognize due to its atypical and insidious presentation [1]. In the largest review to date, Ross and Hu reported only a handful of adolescent cases among 100 total cases, most of which were associated with risk factors such as pelvic surgery, trauma, or immunosuppression [1]. Our case contributes to the small but growing body of literature describing this condition in otherwise healthy adolescent athletes.

The referred pain pattern, absence of direct pubic tenderness, and normal initial radiographs often lead to misdiagnosis or delayed diagnosis. In a comparative study of osteomyelitis pubis versus osteitis pubis, Pauli et al. highlighted the diagnostic overlap and emphasized the importance of considering infection when conservative management fails or systemic signs are present [2]. Our patient’s atypical pain distribution and elevated inflammatory markers raised early suspicion, prompting advanced imaging.

The symphysis pubis is a secondary cartilaginous (amphiarthrodial) joint, non-synovial in nature, located at the midline where the two pubic bones converge. It comprises an interpubic fibrocartilaginous disc interposed between thin articular surfaces of hyaline cartilage covering the opposing pubic bodies [3]. In athletes, particularly those involved in sports requiring frequent twisting and forceful adduction movements, such as football, this region is prone to inflammation and infection as a known complication [1,7,8]. *Staphylococcus aureus *is the pathogen most frequently associated with cases of septic arthritis in athletes, whereas in intravenous drug users, *Pseudomonas aeruginosa* is more commonly implicated [1,2]. The proposed pathogenesis in athletics involves repetitive microtrauma from sporting activities, which predisposes the joint to hematogenous seeding by *Staphylococcus aureus, *a transient organism in the body, leading to subsequent colonization and infection [6].

Historically, there has been considerable diagnostic confusion between osteitis pubis, osteomyelitis, and septic arthritis of the symphysis pubis [1]. Diagnostic delays are common, largely owing to the rarity and atypical location of the condition, compounded by the difficulty in distinguishing it from various urological, gynecological, and abdominal pathologies [9]. MRI is considered the most sensitive modality for detecting the condition, as radiographic and CT changes often appear later in the disease course [1].

In our case, the insidious and atypical presentation contributed to a delay in diagnosis. The pattern of referred pain and absence of localized pubic tenderness further complicated the clinical assessment. MRI proved invaluable in identifying the pathology, whereas plain radiographs did not demonstrate any significant findings. Although aspiration is generally preferred for both diagnostic confirmation and therapeutic management, the small size and anatomical location of the abscesses precluded interventional drainage. Despite this, blood cultures were positive, allowing us to initiate targeted therapy. Previous reports have described similar cases in athletes, particularly those engaging in sports with repeated hip adduction movements, including soccer and hockey [1,6,10]. Dalbavancin, a long-acting lipoglycopeptide antibiotic, has shown efficacy in osteoarticular infections and was effectively used in this case for outpatient management [11,12]. Awareness of this entity can prevent unnecessary investigations and facilitate early, targeted therapy.

## Conclusions

This case highlights septic arthritis of the pubic symphysis as an important, though rare, differential diagnosis in athletes presenting with persistent groin or pelvic pain, even in the absence of overt trauma or classic signs. Sports involving repetitive hip adduction and rotational movements, such as hurling, may predispose individuals to this condition. Early imaging with MRI plays a crucial role in detecting subtle infective changes around the pubic symphysis when plain radiographs are inconclusive. Prompt identification and targeted antimicrobial therapy, guided by microbiological findings, can lead to complete recovery without the need for surgical drainage. Awareness of this entity among clinicians is essential to facilitate early diagnosis and prevent prolonged morbidity.
